# Timing of non-pharmaceutical interventions to mitigate COVID-19 transmission and their effects on mobility: a cross-country analysis

**DOI:** 10.1007/s10198-021-01355-4

**Published:** 2021-07-25

**Authors:** Amit Summan, Arindam Nandi

**Affiliations:** 1 Center for Disease Dynamics, Economics and Policy, 5636 Connecticut Ave NW, PO Box 42735, Washington, DC 20015 USA; 2 The Population Council, New York, NY USA

**Keywords:** COVID-19, Non-pharmaceutical interventions, Social distancing, Pandemic, I12, I18

## Abstract

In the early stages of a pandemic, non-pharmaceutical interventions (NPIs) that encourage physical distancing and reduce contact can decrease and delay disease transmission. Although NPIs have been implemented globally during the COVID-19 pandemic, their intensity and timing have varied widely. This paper analyzed the country-level determinants and effects of NPIs during the early stages of the pandemic (January 1st to April 29th, 2020). We examined countries that had implemented NPIs within 30 or 45 days since first case detection, as well as countries in which 30 or 45 days had passed since first case detection. The health and socioeconomic factors associated with delay in implementation of three NPIs—national school closure, national lockdown, and global travel ban—were analyzed using fractional logit and probit models, and beta regression models. The probability of implementation of national school closure, national lockdown, and strict national lockdown by a country was analyzed using a probit model. The effects of these three interventions on mobility changes were analyzed with propensity score matching methods using Google’s social mobility reports. Countries with larger populations and better health preparedness measures had greater delays in implementation. Countries with greater population density, higher income, more democratic political systems, and later arrival of first cases were more likely to implement NPIs within 30 or 45 days of first case detection. Implementation of lockdowns significantly reduced physical mobility. Mobility was further reduced when lockdowns were enforced with curfews or fines, or when they were more strictly defined. National school closures did not significantly change mobility.

**Supplementary Information:**

The online version contains supplementary material available at 10.1007/s10198-021-01355-4.

## Introduction

As of March 15th, 2021, the novel coronavirus (SARS-COV-2) had infected over 119 million people and caused over 2.6 million confirmed deaths worldwide [1]. Early estimates suggested that the global cost of COVID-19 without containment measures—population-level social distancing along with surveillance and quarantine—would be $9 trillion [2] with a death toll of 40 million [3]. After more than a year, varied government responses and local conditions have contributed to substantial variations in morbidity and mortality across countries. For example, the United States had 19.9% of all global deaths while only containing 4.2% of the global population, while India only had 6% of global deaths while containing 17.5% of the global population [1]. Mortality has been disproportionately concentrated in populations with underlying health conditions such as the elderly and obese [4].

The pharmaceutical industry, supported by the scientific community, multilateral organizations, and local governments, has produced multiple safe and effective vaccines to combat COVID-19 in record time. Although vaccination rollouts have been impressive in several countries, significant challenges remain including vaccine hesitancy, inadequate vaccine supply and delivery, and new COVID-19 strains for which current vaccines may be ineffective [5]. The COVID-19 pandemic has demonstrated that early government response is critical to curbing transmission, reducing mortality, and preventing health systems from being overwhelmed. An understanding of the factors that inform early response and the effects of these factors will be critical to help us respond effectively to future pandemics and to a potential resurgence of COVID-19 due to new virus strains.

At the beginning of the pandemic, national governments took two different approaches to limiting transmission, although some countries later used a combination of both. Countries such as South Korea, Singapore, and Germany used intensive testing, innovative technologies to contract trace, and quarantine and isolation measures to keep cases low, along with moderate social distancing measures [6, 7]. However, the success of such approaches hinges on early implementation [8]. This strategy also requires robust logistics and testing capacity, which many countries lack [9]. Given a basic reproduction number of 2.5 for COVID-19 and a low rate of pre-symptomatic transmission, an isolation and contact tracing approach would require tracing of an estimated 70% of contracts to effectively reduce transmission [10]. Peak infectiousness time for COVID-19 occurs around the time of—including a few days after—symptom onset [11], suggesting that this method alone would not suffice, or would require considerable testing capacity.

The alternative approach was a greater focus on the implementation of non-pharmaceutical interventions (NPIs) that encourage social distancing. NPIs in combination with widespread testing, case detection, contact tracing, and enforcement of quarantine are appropriate where there is widespread community transmission [6, 12]. These measures delayed transmission and flattened the COVID-19 epidemiological curve, and bought governments precious time to prepare for higher caseloads [13–15]. NPIs work best when they are applied as a basket of measures—a rapid review found that quarantine combined with multiple preventive measures such as school closures, travel restrictions, and social distancing had a larger cumulative effect on new COVID-19 cases, transmission rates, and number of deaths than any single intervention alone [16].

The effectiveness of NPIs is a function of when they are implemented, with earlier implementation being more successful in reducing transmission [17]. American cities that implemented multiple NPIs earlier had lower death rates during the 1918 influenza pandemic [18] and similar patterns have been found during the COVID-19 pandemic [19]. However, countries may choose to gradually implement measures or delay implementation altogether to minimize the economic and social costs of lockdowns, or even political costs [20, 21]. These costs include reduced economic growth [22], increased risk of depression and mental health problems due to isolation [23–26], and increased risk of domestic violence [27–29]. Costs may vary by country and within countries; lockdown measures can particularly have disproportionate negative consequences where governments are not able to provide social safety nets [30]. A lack of knowledge about fundamental disease characteristics in the early stages of a pandemic—as with COVID-19—can also delay the most appropriate response [31].

It is important to understand the decision-making process for NPIs to improve resource allocation and create incentives for timely NPI implementation in future pandemics. Although many predictive mathematical models [14, 16, 32, 33] have simulated the effect of NPIs on COVID-19, there is limited evidence on the actual effect of these interventions globally and the decision models that influenced their implementation. We examined country-level health systems capacity, epidemiological, and socioeconomic characteristics associated with delay in the implementation of three NPIs: national school closure, global travel ban, and country-wide lockdowns, during the period January 1st, 2020, to April 29th, 2020, and the effect of these NPIs on population mobility.

## Data and methods

### Data

Daily data on the number of COVID-19 cases were collected from the European Center of Disease Prevention and Control (ECDPC) [34]. Our data on NPIs were drawn from two datasets—the ACAPS (Assessment Capacities Project) COVID-19 government measures dataset [35] (May 1, 2020 release) and a dataset constructed by the University of Oxford [36] (April 29, 2020 release). Both datasets contain information on global, country-level COVID-19-related policy interventions, and implementation dates. We scrutinized the data and their sources and checked for consistency across the two datasets to ensure accuracy of the data. We considered three interventions: national school closure, national lockdown, and global travel ban. We did not include countries where measures were implemented only at the sub-national level (e.g., province or city).

We used Google’s recent mobility reports which track mobile device location data for over 130 countries as measures of social mobility and physical distancing [37]. These reports have been available since mid-February of 2020 and have been updated on a weekly basis to aid policy makers. The reports show trends in how visits and length of stay in different locations change compared to the median value, for the corresponding day of the week, during the period of January 3, 2020 to February 6, 2020. Google identifies six location types for which mobility data are tracked: retail and recreation, grocery and pharmacy, parks, transit stations, workplaces, and residential.

Our control variables included health systems, epidemiological, and socioeconomic characteristics which can affect disease transmission rates, and cost and benefits of NPI implementation. The following variables were collected from the World Bank database [38]: population density, percentage of the population under the age 15, and total population. We included eight sub-regions of the world to capture cultural or geographical factors that may affect the response to COVID-19 and country income category (low, lower middle, upper middle, and high) from the World Bank. Additionally, we included a measure of government regime type from the Center for Systemic Peace [39]. This variable varied from − 10, indicating an autocracy, to 10, indicating a full democracy. We also included the day the first case was detected in the country as an additional control variable. As a measure of health systems capacity related to pandemics, we used the global health security index score—developed by the Nuclear Threat Initiative and the Johns Hopkins Center for Health Security—which uses 140 variables related to six categories: prevention, detection and reporting, rapid response, health system, compliance with international norms, and risk environment, to develop a country score between 0 and 100, where a higher score indicates a greater level of pandemic preparedness [40]. Finally, we included temperature change from February 15th to March 1st in the country’s capital city using data from the National Aeronautics and Space Administration [41]. Weather changes can affect mobility which could affect disease transmission and policy decisions.

### Methods

#### Outcomes

We examined three NPIs: lockdown, global travel ban (border closure to non-essential travel), and school closure. All three measures curb social interaction among individuals. Although the evidence on the effect of travel bans [42–45] and school closures [46–49] on transmission delay and spread is mixed, we included both of these NPIs because of their widespread implementation globally. We focused on national-level interventions as sub-national data may not always be available or complete.

We defined lockdown as the closure of all non-essential businesses and allowance of leaving home only for essential activities. The definition of essential activities and businesses may vary by country. For example, some countries closed all retail stores, recreational business and areas, and workplaces that may be at high risk for transmission, and recommended all others to work from home, and only go to work if *absolutely* necessary. What is considered absolutely necessary may vary by country, by employer, or to an individual. Some countries allowed for exercise outside of the home or leaving the home to get ‘fresh air’ for a limited time. To distinguish between the intensity of the lockdown, we created a measure of strict lockdown. A strict lockdown was considered one in which all businesses were closed except for those deemed essential, e.g., pharmacies, grocery stores, financial institutions, healthcare, and food production, and individuals were only allowed to leave their homes to work at an essential business, buy groceries, or take care of medical needs including accessing care or providing care to family members.

We also considered lockdown to be strict if a lockdown was accompanied by one of the following: (1) a curfew which allowed individuals to engage in sanctioned activities outside the home at specific time intervals, (2) a fine which would be issued if individuals were not complying with lockdown measures, and (3) additional military presence to enforce lockdown measures. We will refer to a non-strict lockdown as a normal lockdown henceforth. We only considered lockdowns that had a minimum length of 72 h, which excluded some countries that, for example, implemented measures during weekends or extended weekends only. We used the earliest type of lockdown implemented for a country, resulting in one observation per country. This is important because many countries started with a normal lockdown and slowly transitioned into a strict lockdown or vice versa.

We created three binary variables that considered if a lockdown, strict lockdown, or national school closure was implemented within a certain time frame. We also created continuous variables that measured the delay in implementation of either a lockdown, school closure, or global travel ban after first, fifth, or tenth case detection. The outcome variables are described in Table 1. The binary variables of NPI implementation only considered countries that had implemented the policy within 30 or 45 days or had a minimum of 30 or 45 days pass since first case detection, respectively. This is important, because (1) our focus is on early implementation of NPIs and (2) this excludes countries where COVID was reported substantially later, as these countries would be influenced by political and economic considerations that we may not be able to capture in our analysis. Our final dataset had 122, 123, 119, and 121 countries available for the binary variables of school, lockdown, strict lockdown, and air travel NPI implemented within 45 days of first case detection, respectively.

**Table 1:** Summary of outcomes variables

Variable	Policy	Description	Maximum delay (days) in implementation since first case detection
School30	National school closure	Coded 1 if policy implemented within specified time period. Coded 0 if days since first case were at least the length of the maximum specified time period and the NPI was not implemented	30
School45			45
Lockdown30	National lockdown (closure of all non-essential businesses and public areas; residents strongly recommended to stay at home and leave only when absolutely necessary)		30
Lockdown45			45
Lockdown_strict30	National lockdown (strict:): (1) only essential business open, not allowed to leave home except for essential needs (food, work in essential business, healthcare related needs), (2) *normal* lockdown + curfew (limited times) for leaving home to perform essential activities, (3) normal lockdown + fines if lockdown policies or violated, or (4) normal lockdown + military presence to enforce lockdown		30
Lockdown_strict45			45
Delay1_30	Lockdown, national school closure, or global travel ban* (on non-essential travel)	Delay in days in implementation of one of the policies within the specified time period since first case detection	30
Delay1_45			45

*Any country in the European Union was considered to have implemented a global travel ban if it had implemented a ban to at least all non-Schengen zone countries

The final set of variables measured the change in mobility. The main outcome variable was the percentage change in mobility from 1 day before to 2 days after the implementation of an NPI for each of the six locations for which Google reports data. We focused on a short post-intervention follow-up period for our main analysis, because the probability that a country changes recently implemented measures or implements new measures increases with time and may make it difficult to isolate the effects of the intervention of interest. For additional sensitivity analysis, we looked at the change in mobility from 1 day before to 6 days after NPI implementation.

#### Estimating associations with delay in policy implementation

To estimate the association of country characteristics with delay in implementation, we employed three methodologies. The variables measuring delay in implementation contain discrete values and are bounded below by 0 and above by 30 or 45—the maximum delay in days until implementation of an NPI after case detection, as described in Table 1. As our outcome was bounded, we standardized the delay variable to be contained within the interval [0, 1], by dividing the outcome variable by 30 or 45 days depending on the latest implementation day for countries to be considered for analysis. Then, we employed fractional logit regression and two additional models for sensitivity analysis—fractional probit and beta regression. The beta distribution requires values to be bounded between (0, 1) and cannot include boundary values; therefore, we added epsilon (1^–10^) to transform delay values of 0 and subtracted epsilon from delay values of 1. This model was regressed on the type of NPI, region, income level, health preparedness score, log of population density, log of population, measure of government regime type, percentage of population under 15, and temperature change between February 15th, 2020 and March 1st, 2020. Standard errors were clustered at the country level.

#### Propensity score matching

We used propensity score matching (PSM) to estimate the effect of NPIs on mobility outcomes. PSM is a widely used quasi-experimental approach used to analyze the effects of interventions in non-experimental data. In observational settings, assignment to intervention or control groups, or self-selection into a group is not random. Assignment is typically correlated with several variables and a simple comparison of outcomes between intervention and control groups may produce biased estimates. PSM homogenizes the two groups by matching each intervention observation with one or more similar control observations. The difference in outcome between the two matched groups is known as the average treatment effect on the treated (ATT).

In our analysis, there may be statistically significant differences in epidemiological, economic, and political characteristics between countries that decided to implement an NPI in a timely manner (intervention countries) and those that did not (control countries). These systematic differences could bias ordinary least square estimates of associations between NPI intervention and mobility. PSM compares each country that implemented an NPI with a country that did not implement the NPI, but had a similar probability of implementing it based on observed characteristics. The difference in mobility would be attributable to the NPI intervention if the distributions of unobservable factors that affect NPI implementation were random between control and intervention countries.

We employed a probit model to regress the binary indicator of whether a country implemented NPI on a set of covariates which included the region, income level, health preparedness score, log of population density, measure of government regime type, log of total population, young population as percentage of total population, date of first case detection, and temperature change. Based on the predicted probability (propensity score) from this regression, we matched intervention countries with control countries. We used one-to-one nearest-neighbor matching with replacement. For sensitivity analyses, we matched observations to the nearest three neighbors and employed the kernel matching algorithm. In all models, common support was imposed, where observations whose propensity scores did not overlap between the two groups were discarded.

We investigated the quality of matching between the groups in three ways. First, we looked at difference in mean and median percentage bias across all matching variables before and after matching. A reduction in bias indicated the matching procedure had made the control and intervention groups more comparable. Second, we considered the *p* value of the likelihood ratio test of joint significance of all matching variables on the propensity score. Finally, we evaluated the pseudo-*R*^2^ of this model. A higher *p* value after matching or lower pseudo-*R*^2^ indicates a reduction in systematic differences in variables. All analysis was conducted using Stata version 14.2 and we considered *p* < 0.05 for statistical significance.

#### Parallel trends test

The parallel trends test is an important measure of methodological validity in difference-in-difference analyses. If the parallel trends tests were to be satisfied, mobility rates in countries that implemented NPI and those that did not implement NPI should follow a similar trend prior to NPI introduction. We tested if trends in mobility between NPI and non-NPI countries were statistically indistinguishable between February 15th, 2020 and March 1st, 2020. February 15th was the earliest day for which mobility data were available from Google.

We tested for parallel trends in two ways [50]. First, we estimated country fixed effect regression models of time spent in residence from February 15th, 2020 to March 1st, 2020 on the set of control variables used in the PSM model. However, instead of temperature change in capital city between February 15th and March 1st, we used daily temperature as a control variable. Then, we examined if the time trends of the estimated residual error terms of these models were parallel across countries that implemented a lockdown within 30 days and those that did not. Second, we regressed time spent in residence on day identifiers, binary indicators of whether a country implemented a lockdown within 30 days, and interaction terms of the day and lockdown identifiers. If the estimated regression coefficients of the interaction terms were not statistically significant, the parallel trends assumption would be satisfied, i.e., trends in mobility would be similar between NPI and non-NPI countries leading up to the implementation of NPIs.

## Results

### Summary characteristics of data

Table 2 shows the factors associated with delay in implementation of NPIs for countries after detecting their first, fifth, and tenth COVID-19 case, excluding countries that did not implement NPIs within 30 or 45 days of case detection. Mean delay for implementation of nationwide school closure was shortest among the three interventions—an average of 13 days after first case detection. This was followed by international air travel restrictions at 18 days, and national lockdowns at 21 days after the first case. The mean delay to implementation of any measure on average was highest for South Asia and the East Asia and Pacific regions, while it was lowest for the Sub-Saharan Africa and Latin America and Caribbean regions. We divided the continuous variables into categorical variables below and above their median values for ease of interpretation. Countries with lower income; less democratic political systems; lower levels of health preparedness; and older, smaller, and less denser populations implemented measures earlier on average. The difference in delay in NPI implementation after first case detection was greatest between countries with low and high health preparedness levels, with an average of 22 days for countries above and 10 days for countries below the median score; young population as proportion of total population, with an average of 11 days for countries above and 22 days for countries below the median young population; and between low-income and high-income countries, with an average of 9 days in delay relative to 22 days, respectively. The difference in delay between these country types remained when delay was measured after fifth and tenth case detection; however, the magnitude of the differences decreased.

**Table 2:** Mean delay (days) in implementation of non-pharmaceutical intervention by country characteristics

	Delay1	Observations	Delay5	Observations	Delay10	Observations
Intervention type						
Air	17.57	109	11.49	105	10.37	98
Lockdown	21.04	79	14.79	78	12.52	73
School	13.19	137	7.27	135	5.88	129
Region						
East Asia & Pacific	39.73	30	36.48	27	32.19	27
Europe & Central Asia	19.35	100	12.32	100	10.26	100
Latin America & Caribbean	9.05	60	5.93	60	4.19	58
Middle East & North Africa	15.34	41	9.61	41	7.34	41
North America	22.67	3	18.00	3	10.33	3
South Asia	37.58	12	8.92	12	7.90	10
Sub-Saharan Africa	7.16	79	2.84	75	2.28	61
Income level						
Low income	8.64	44	1.95	40	1.42	31
Lower middle income	13.33	67	5.39	64	4.38	60
Upper middle income	16.19	102	11.38	102	9.88	97
High income	21.96	112	15.69	112	12.71	112
Political system*						
Below median	15.49	144	9.66	137	8.61	126
Above median	18.52	154	11.91	154	9.96	147
% of population under 15						
Below median	21.93	164	15.60	164	12.98	164
Above median	11.21	153	5.14	146	4.17	128
Log of population						
Below median	12.55	130	7.64	129	6.61	118
Above median	19.43	193	12.59	187	10.60	180
Log of population density						
Below median	12.55	168	7.44	165	5.94	156
Above median	21.74	149	14.34	147	12.75	138
Health preparedness score						
Below median	9.56	130	4.15	123	3.17	107
Above median	21.98	182	15.05	182	12.61	180
Temperature change, Feb. 15th to Mar. 1st 2020						
Below median	17.11	152	10.86	146	9.89	135
Above median	16.42	162	10.33	161	8.27	154

*Delay1* delay in days in implementation of one of three NPIs after detection of first case. If policy was implemented before the 1st, 5th, or 10th case was detected, a delay value of 0 was used

*A higher score indicates a more democratic political system

### Delay in NPI implementation

Table 3 shows the results from the fractional logit, fractional probit, and beta regression models for the number of days it took to implement an NPI given that the country implemented the measure within 30 or 45 days. The response variable was transformed to be between 0 and 1, such that each 0.1 interval would correspond to 3 days if the policy was implemented within 30 days and 4.5 days if the policy was implemented within 45 days, since first case detection, respectively. According to five of the models, countries implemented national school closures (odds ratio [OR] 0.531, 95% confidence interval [CI] 0.392–0.718, *p* value < 0.01; model 1) faster relative to air travel restrictions. The Latin American and Caribbean region had a shorter delay in implementation of NPIs in five models (OR 0.051, CI 0.003–0.883, *p* value < 0.05; model 1) relative to North America. The log of total population was associated with greater delay in implementation of all NPIs (OR 1.245, CI 1.068–1.451, *p* value < 0.01; model 1). Countries with greater health preparedness scores (OR 1.024, 1.002–1.046, *p* value  < 0.05; model 1) had larger delays in implementation of NPIs in three models (two of the three models for NPI implemented within 30 days of first case detection).

**Table 3:** Country characteristics and delay in non-pharmaceutical intervention implementation

Maximum delay in implementation (days)	30			45		
Model	1	2	3	4	5	6
	Fractional logit	Fractional probit	Beta regression	Fractional logit	Fractional probit	Beta regression
Intervention (Air = 0)						
Lockdown	1.2	1.127	2.232**	1.089	1.062	1.739**
	(0.888—1.623)	(0.939–1.353)	(1.418–3.512)	(0.875–1.356)	(0.929–1.213)	(1.150–2.629)
School	0.531**	0.684**	0.718	0.622**	0.756**	0.704
	(0.392–0.718)	(0.570–0.821)	(0.432–1.195)	(0.481–0.804)	(0.649–0.880)	(0.444–1.115)
Region (North America = 0)						
East Asia & Pacific	0.21	0.471	1.83	0.106^+^	0.324**	1.536
	(0.013–3.428)	(0.090–2.474)	(0.703–4.766)	(0.007–1.521)	(0.166–0.631)	(0.629–3.753)
Europe & Central Asia	0.092^+^	0.284	0.816	0.065*	0.243**	0.965
	(0.006–1.517)	(0.053–1.510)	(0.331–2.011)	(0.005–0.867)	(0.124–0.475)	(0.424–2.195)
Latin America & Caribbean	0.051*	0.197^+^	0.428*	0.032*	0.160**	0.409*
	(0.003–0.883)	(0.036–1.077)	(0.190–0.963)	(0.002–0.494)	(0.086–0.298)	(0.198–0.842)
Middle East & North Africa	0.164	0.405	1.777	0.080^+^	0.275**	1.684
	(0.009–3.158)	(0.069–2.375)	(0.712–4.434)	(0.005–1.305)	(0.149–0.507)	(0.847–3.348)
South Asia	0.202	0.465	1.242	0.109	0.339	1.945
	(0.007–6.004)	(0.062–3.498)	(0.260–5.925)	(0.004–2.688)	(0.339–0.339)	(0.452–8.364)
Sub-Saharan Africa	0.065^+^	0.234		0.044*	0.194**	
	(0.003–1.294)	(0.039–1.381)		(0.003–0.735)	(0.111–0.341)	
Income level (low income = 0)						
Lower middle income	1.092	1.045	2.644*	1.068	1.029	2.473*
	(0.555–2.151)	(0.706–1.547)	(1.093–6.396)	(0.591–1.930)	(0.740–1.432)	(1.076–5.686)
Upper middle income	2.025	1.508	3.284*	1.901^+^	1.438^+^	2.981*
	(0.858–4.777)	(0.912–2.493)	(1.153–9.352)	(0.925–3.907)	(0.956–2.162)	(1.181–7.523)
High income	2.317^+^	1.639	3.181^+^	2.383^+^	1.653^+^	3.340*
	(0.854–6.284)	(0.909–2.956)	(0.965–10.481)	(0.981–5.789)	(0.994–2.748)	(1.150–9.698)
Political regime*	1.018	1.011	1.009	1	1	1.006
	(0.986–1.051)	(0.992–1.030)	(0.976–1.042)	(0.972–1.029)	(0.984–1.017)	(0.976–1.038)
% of population under 15	1.008	1.004	0.975	0.995	0.997	0.971
	(0.975–1.042)	(0.984–1.025)	(0.933–1.018)	(0.965–1.027)	(0.978–1.016)	(0.933–1.011)
Log of population	1.245**	1.140**	1.447**	1.336**	1.183**	1.477**
	(1.068–1.451)	(1.040–1.251)	(1.200–1.744)	(1.159–1.541)	(1.088–1.287)	(1.255–1.737)
Log of population density	1.028	1.014	1.121	1.02	1.008	1.108
	(0.871–1.213)	(0.916–1.122)	(0.923–1.362)	(0.883–1.178)	(0.926–1.098)	(0.925–1.327)
Health preparedness score	1.024*	1.014*	1.026^+^	1.016^+^	1.009^+^	1.025*
	(1.002–1.046)	(1.001–1.027)	(0.999–1.055)	(0.998–1.034)	(0.998–1.020)	(1.002–1.050)
Mean temperature change, Feb. 15 to Mar. 1	0.971^+^	0.982^+^	0.969	0.975	0.985	0.968
	(0.941–1.001)	(0.964–1.000)	(0.931–1.008)	(0.944–1.007)	(0.967–1.004)	(0.931–1.007)
Observations	241	241	241	257	257	257
Pseudo * R*^2^	0.1	0.1		0.09	0.09	

Outcome variable is the number of delays in lockdown on a scale of (0, 1) where 0 corresponds to 0 day delay and 1 corresponds to the maximum allowed delay (30 or 45 days). If policy was implemented before the first case was detected, a delay value of 0 was used. Results reported in odds ratio. Standard errors clustered at country level. 95% confidence intervals in parentheses. *A higher score indicates a more democratic political system

^+^*p* < 0.1, *p < 0.05, **p < 0.01

### Likelihood of NPI implementation

Table 4 shows the likelihood of implementing school closures and lockdowns before 30 and 45 days after detecting the first case. The likelihood of implementing a normal lockdown or strict lockdown was not significantly associated with the country’s region. Countries in the East Asia and Pacific region were significantly less likely to implement school closures within 30 days of first case detection, while Middle East and North African countries were significantly more likely to enforce school closures, relative to North American countries. Upper middle-income countries were more likely than low-income countries to implement any of the three NPIs within 30 days of first case detection. More democratic countries were more likely to implement a lockdown (OR 1.055, CI 1.006–1.107, *p* value  < 0.05; model 1), but no significant association was found between political regime and implementation of a strict lockdown. The later the first case was detected the more likely the country was to implement any NPI within 30 or 45 days of first case detection. Countries that were denser were more likely to implement a lockdown, including a strict lockdown (OR 1.268, CI 1.022–1.571, *p* value  < 0.05; model 3), while countries with larger populations were more likely to implement only a national school closure within 45 days of first case detection.

**Table 4:** Probability of implementing school and lockdown intervention by timeliness

Model	1	2	3	4	5	6
Intervention	Lockdown	Lockdown	Lockdown Strict	Lockdown Strict	School	School
Maximum implementation delay (days)	30	45	30	45	30	45
Region (North America = 1)						
East Asia & Pacific	0.703	1.695	0.57	0.894	0.287*	0.461
	(0.193–2.554)	(0.428–6.710)	(0.169–1.928)	(0.192–4.166)	(0.089–0.928)	(0.113–1.882)
Europe & Central Asia	1.283	2.404	0.561	0.761	0.956	0.994
	(0.357–4.616)	(0.650–8.892)	(0.178–1.765)	(0.227–2.549)	(0.233–3.928)	(0.178–5.563)
Latin America & Caribbean	0.88	1.283	0.679	0.768	0.613	0.519
	(0.312–2.481)	(0.412–3.996)	(0.250–1.845)	(0.261–2.258)	(0.203–1.854)	(0.157–1.716)
Middle East & North Africa	1.92	2.058	1.256	1.145	7.057**	
	(0.606–6.083)	(0.662–6.397)	(0.428–3.685)	(0.378–3.467)	(1.708–29.149)	
South Asia	0.603	0.559	1.008	0.78	1.507	1.545
	(0.146–2.498)	(0.133–2.350)	(0.266–3.823)	(0.197–3.098)	(0.377–6.021)	(0.362–6.593)
Income group (low income = 1)						
Lower middle income	2.849*	1.796	2.233^+^	1.512	1.792	0.815
	(1.108–7.331)	(0.676–4.775)	(0.865–5.763)	(0.573–3.992)	(0.658–4.879)	(0.241–2.755)
Upper middle income	5.261**	3.025^+^	6.111**	3.385^+^	5.173*	1.936
	(1.580–17.516)	(0.819–11.170)	(1.839–20.310)	(0.990–11.576)	(1.443–18.546)	(0.479–7.822)
High income	5.107*	3.241	3.135	1.796	7.147*	5.975^+^
	(1.149–22.700)	(0.680–15.456)	(0.693–14.186)	(0.399–8.081)	(1.394–36.633)	(0.768–46.454)
Day of first case	1.070**	1.060**	1.052**	1.043**	1.069**	1.083**
	(1.041–1.100)	(1.032–1.089)	(1.027–1.078)	(1.014–1.073)	(1.038–1.101)	(1.043–1.124)
Political regime*	1.055*	1.056*	1.016	1.019	1.039	1.026
	(1.006–1.107)	(1.005–1.110)	(0.971–1.062)	(0.970–1.071)	(0.989–1.092)	(0.963–1.094)
% of population under 15	0.972	0.984	0.992	0.989	1.027	1.003
	(0.913–1.035)	(0.922–1.051)	(0.934–1.052)	(0.931–1.051)	(0.955–1.104)	(0.917–1.097)
Log of population	1.199	1.183	1.032	1.019	1.247^+^	1.408*
	(0.960–1.497)	(0.943–1.485)	(0.852–1.250)	(0.832–1.249)	(0.982–1.585)	(1.017–1.948)
Log of population density	1.348**	1.415**	1.268*	1.266*	0.906	1.008
	(1.076–1.689)	(1.122–1.786)	(1.022–1.571)	(1.034–1.551)	(0.703–1.169)	(0.741–1.370)
Health preparedness score	0.99	0.98	1.024	1.014	0.999	0.975
	(0.956–1.024)	(0.947–1.014)	(0.991–1.058)	(0.981–1.048)	(0.962–1.037)	(0.929–1.024)
Mean temperature change, Feb. 15 to Mar. 1	1.029	1.02	1.015	1.008	1.006	1.026
	(0.978–1.083)	(0.968–1.075)	(0.963–1.069)	(0.957–1.061)	(0.951–1.064)	(0.960–1.097)
Observations	147	134	147	131	147	123
Pseudo *R*^2^	0.269	0.261	0.179	0.147	0.35	0.441

Results reported in odds ratio. Robust standard errors. 95% confidence intervals in parentheses

*A higher score indicates a more democratic political system

^+^*p* < 0.1, *p < 0.05, **p < 0.01

### Parallel trends test results

Figure A1 in the online supplementary appendix shows the time spent in residential location between February 15th, 2020 and March 1st, 2020 for countries that implemented a lockdown within 30 days of first case detection relative to countries which did not implement the NPI. Figure A2 in the online supplementary appendix shows the residual of time spent in residence between countries that implemented NPIs and those that did not. As demonstrated in these graphs, time trends of mobility and the residual were similar for NPI intervention and non-intervention countries. Figure A3 in the online supplementary appendix presents the estimated coefficients (with 95% confidence intervals) of the interaction terms between year and NPI indicators in the regression of time spent in residence. Leading up to the implementation of NPIs, there was no statistical difference between mobility rates in intervention and control countries. Based on these figures, we assume that the parallel trends assumption is satisfied.

### Effect of NPIs on mobility

Table 5 shows the effects of the NPIs on mobility, from 1 day before to 2 days after policy implementation. Location data suggest that there was a significant reduction in time spent outside the house from the implementation of lockdowns regardless of when they were implemented. A strict lockdown decreased mobility to a greater degree than a normal lockdown. When implemented within 30 days of first case detection, time spent in residential areas increased by 6.67% (CI 3.58–9.75%, *p* value  < 0.01) under a normal lockdown relative to an increase of 9.40% (CI 6.08–12.72%, *p* value  < 0.01) in a strict lockdown. Small percentage changes in time spent in residence will indicate large changes in total absolute time spent in residence given the higher baseline amount of time spent at home. The greatest change in time spent in a location was for time spent in grocery and pharmacy, where there was a reduction of 30.98% (CI 20.51–41.44%, *p* value  < 0.01) when a strict lockdown was implemented within 30 days of first case detection. Table A1 in the online supplementary appendix shows the effects of NPIs with longer follow-up periods (6 days after NPI implementation). There were no substantial differences between the short and long follow-up period results; however, for school closures implemented within 45 days, there was an increase in time spent in residence of 5.04% (CI 0.34–9.74%, *p* value  < 0.05). The PSM results for matching to nearest three neighbors and kernel matching results are shown in the online supplementary appendix in Tables A2 to A4. The coefficients do not vary substantially, and the results are not sensitive to the matching algorithm used.

**Table 5:** Propensity score matching results on change in mobility from the implementation of non-pharmaceutical intervention

Measure	Latest day of implementation	Category	Change in mobility (%)	Confidence interval	*R*^2^	Observations
Lockdown	30	Grocery and pharmacy	− 19.82**	(− 29.12 to − 10.53)	0.14	112
		Parks	− 13.96**	(− 21.91 to − 6.01)	0.099	112
		Residential	6.67**	(3.58–9.75)	0.145	110
		Retail and recreation	− 16.25**	(− 23.42 to − 9.07)	0.154	113
		Transit stations	− 15.84**	(− 21.55 to − 10.13)	0.215	112
		Workplace	− 15.41**	(− 21.62 to − 9.20)	0.179	113
	45	Grocery and pharmacy	− 26.38**	(− 36.16 to − 16.61)	0.218	105
		Parks	− 20.06**	(− 26.96 to − 13.17)	0.244	105
		Residential	6.62**	(3.48–9.77)	0.147	103
		Retail and recreation	− 23.21**	(− 30.88 to − 15.54)	0.259	105
		Transit stations	− 19.77**	(− 25.87 to − 13.66)	0.286	105
		Workplace	− 11.74**	(− 18.07 to − 5.41)	0.116	105
Lockdown strict	30	Grocery and pharmacy	− 30.98**	(− 41.44 to − 20.51)	0.238	112
		Parks	− 19.41**	(− 28.21 to − 10.61)	0.148	112
		Residential	9.40**	(6.08–12.72)	0.227	109
		Retail and recreation	− 25.58**	(− 33.39 to − 17.76)	0.275	113
		Transit stations	− 21.70**	(− 28.13 to − 15.27)	0.289	112
		Workplace	− 21.69**	(− 28.58 to − 14.79)	0.259	113
	45	Grocery and pharmacy	− 30.65**	(− 40.96 to − 20.34)	0.256	103
		Parks	− 17.68**	(− 25.73 to − 9.62)	0.158	103
		Residential	10.77**	(7.19–14.36)	0.262	102
		Retail and recreation	− 23.49**	(− 31.59 to − 15.38)	0.247	103
		Transit stations	− 20.88**	(− 27.71 to − 14.06)	0.267	103
		Workplace	− 24.67**	(− 33.62 to − 15.73)	0.229	103
School	30	Grocery and pharmacy	− 3.74	(− 13.79 to 6.30)	0.006	94
		Parks	− 7.87^+^	(− 16.90 to 1.15)	0.032	94
		Residential	1.78	(− 1.88 to 5.44)	0.01	92
		Retail and recreation	− 4.5	(− 12.22 to 3.22)	0.013	106
		Transit stations	− 4.73	(− 11.33 to 1.87)	0.022	94
		Workplace	− 3.17	(− 11.03 to 4.70)	0.006	106
	45	Grocery and pharmacy	− 5.48	(− 15.63 to 4.67)	0.012	96
		Parks	1.4	(− 8.11 to 10.91)	0.001	96
		Residential	0.37	(− 3.47 to 4.21)	0	94
		Retail and recreation	− 6.31	(− 14.89 to 2.27)	0.022	96
		Transit stations	− 3.68	(− 10.55 to 3.19)	0.012	96
		Workplace	− 3.26	(− 10.70 to 4.17)	0.008	96

Change in mobility from 1 day before to 2 days after intervention. Results from propensity score matching using one-to-one nearest-neighbor matching and imposing common support. 95% confidence intervals in parentheses

^+^*p* < 0.1, *p < 0.05, **p < 0.01

Tables A5 to A7 in the online supplementary appendix show measures of balance and the ability of the matching model to reduce systematic differences between the intervention and control variables to make both groups comparable for analysis. Balancing tests are shown for all location categories, because some countries were missing location data on some days, which may make matching results different across models for each location. For normal and strict lockdowns, the PSM procedure decreased differences between the intervention and control groups after matching. There were substantial reductions in mean and median percentage difference between control and intervention groups on matching variables. The *p* value of the joint significance test was higher and insignificant in the matched sample and the pseudo-*R*^2^ was substantially lower. For measuring the effect of school closure implemented before 30 days, balance was achieved between the intervention and control groups.

## Discussion

COVID-19 has presented an unprecedented challenge to policymakers globally. Local health systems, the global economy, and society at large have been under tremendous pressure in effectively responding to the pandemic. Decisions to implement restrictions on movement or limit the availability of services have faced resistance at times, but reduced disease transmission, morbidity, and mortality. The value of NPIs is greatest if they are implemented early. Our analysis showed that there are health capacity, socioeconomic, and epidemiological factors that may determine which NPIs a country implements and the timing of those interventions. Furthermore, we found that lockdowns reduce mobility and could be even more effective when backed by measures such as curfews or fines. Weak stay-at-home orders that merely suggest working at home and only leaving when absolutely necessary reduce mobility, but at lower rates than orders that strictly define when an individual can leave home. The economic and social costs of stricter lockdown measures should be weighed against the decreases in disease transmission.

Many of our other findings were consistent with theory. Countries with higher population density, higher income, and later first case detection were more likely to implement NPIs. Population density is a risk factor for transmission, where more crowding and contact can increase the rate of transmission, while delayed arrival of COVID-19 may give countries time to prepare for policies and garner public support for interventions. Higher income countries may be able to absorb the costs of NPIs and provide social safety nets for their citizens. National lockdowns may be infeasible in poorer countries where support systems are inadequate, and large segments of the population are daily wage workers.

For countries that implemented lockdowns, those with greater health security preparedness had a longer delay to implementation. The goal of NPIs is to reduce peak prevalence to ensure that hospitals have the necessary equipment and health workers to handle patient load. Therefore, a country with greater health systems capacity may delay implementation of NPIs, because they can respond to a higher number of peak infections without overwhelming the health system. Larger countries were found to have longer delays in implementation which may be due to logistical challenges in covering a very large population under an NPI.

We found that democratic countries were more likely to implement lockdowns. Existing legal frameworks and political systems can determine a country’s ability to implement measures nationally in a timely manner, including the ability to declare a national emergency; allocate resources toward diagnostics, prevention, and treatment; or issue travel restrictions [51, 52]. Although autocratic regimes can generally implement measures more quickly relative to more democratic political systems, the latter may centralize powers within the federal government to act equally rapidly during national crises [53]. Furthermore, democratic governments are more accountable to their constituents, and therefore may take more proactive measures to protect their health and may already have better health infrastructure in place to meet this goal [54].

Our results confirm that country-specific response and adherence to international guidance on NPIs depends on complex political and economic factors, and public health systems capacity [55]. Incentives should be created, so that countries with greater resources and health systems capacities do not significantly delay the implementation of NPIs to limit global transmission early. Pandemic preparedness investments, related to early detection systems, laboratory diagnostic capacity, surveillance, and general health systems capacity should be made globally to increase capacity to combat future disease outbreaks [56]. These should meet international benchmarks, which should be continuously reviewed and revised. During a pandemic information, sharing and research should also be prioritized and incentivized to help limit transmission and the health toll, especially in countries where the disease outbreak initially spreads as these countries lack vital knowledge and data on how to best contain the outbreak in the most cost-effective manner.

The effect of school closures on COVID-19 transmission and mortality has been extensively discussed in the literature [46–49]. There is a concern that school closures may have adverse consequences. Children outside of school, combined with lack of daycare options, may require caregivers outside of the home such as relatives to travel to a child’s home to care for them which may cause disease transmission. Conversely, parents or guardians may not go to work or work from home to care for children. Some of these caregivers may also be healthcare workers, decreasing the critical supply of healthcare workers during a pandemic [49]. Furthermore, students may continue to engage in social and physical contact outside of schools even after closures. Our main model results showed no significant effects of school closures on physical mobility and this remains an area for further research.

There are some limitations to our analysis. Lack of testing capabilities and pandemic preparedness may have created a lag in detection of cases and caused a measurement error in our data. Furthermore, in some countries, stigmatization or overcrowding of health facilities may make it difficult to track disease arrival and spread as individuals fail to report health symptoms to authorities [22]. Second, the Google mobility reports are not perfect measures of mobility. Although smart phone use has significantly increased in past years, poorer countries or older populations may be less likely to use smartphones, resulting in measurement error in mobility. However, these may be the best measures of mobility currently available. Third, we have excluded countries that implemented sub-national NPIs in our analysis due to data collection challenges. It can be argued that for a pandemic such as COVID-19, a patchwork response is not adequate to prevent transmission. However, we recognize that logistical, social, and political considerations may not allow for national NPI implementation. Future analysis should focus on the effects of sub-national policies on mobility and disease transmission. Finally, although we made efforts to distinguish between intensities of social distancing measures by looking at a normal lockdown relative to a strict lockdown, there are challenges in categorizing different types of NPIs due to the large variation in NPIs across countries. Our analysis also ignored complementary measures that were implemented such as restrictions on public and private transit or economic measures that compensated individuals who were unable to work during a lockdown.

NPI implementation and its timing are based on an assessment of the potential health, economic, and social costs and benefits of different policy options. However, pandemics such as COVID-19 make the implementation of such measures a global public good. It is important to consider what constraints countries face and how resources can be better allocated to improve timely implementation of these measures. We provide evidence that these lockdowns, especially those backed by curfews and fines, or stricter stay-at-home orders can reduce physical mobility which may reduce transmission in the early stages of a pandemic. Future research should analyze the economic and social costs of these measures, and how different variations of these measures can maximize reduction in disease transmission while minimizing costs.

## Supplementary Information

Below is the link to the electronic supplementary material.

Supplementary file1 (DOCX 145 kb)

## Data Availability

Data are publicly available from published sources.
