# Supplementary material for: Timing of non-pharmaceutical interventions to mitigate COVID-19 transmission and their effects on mobility: a cross-country analysis
Source: Eur J Health Econ. 2021 Jul 25;23(1):105–17. doi: 10.1007/s10198-021-01355-4 (PMC8310614; doi:10.1007/s10198-021-01355-4)
Supplement: Supplementary file 1 — Supplementary file1 (DOCX 145 kb) [file 10198_2021_1355_MOESM1_ESM.docx]

**Supplementary appendix**

**Timing of non-pharmaceutical interventions to mitigate COVID-19 transmission and their effects on mobility: A cross-country analysis**

**Journal**: The European Journal of Health Economics

**Amit Summan**

Center for Disease Dynamics, Economics & Policy,

5636 Connecticut Ave NW, PO Box 42735, Washington, DC 20015

Phone: (+1) 202-939-3300

*Corresponding author email*: [summan@cddep.org](mailto:summan@cddep.org)

**Arindam Nandi**

The Population Council,

New York, NY;

Center for Disease Dynamics, Economics & Policy,

Washington, DC

Email: [nandi@cddep.org](mailto:nandi@cddep.org)

Figure A1: Time trends of time spent in residence between lockdown and non-lockdown countries, February 15^th^, 2020 to March 1^st^, 2020

Note: N=132 countries; 84 non-NPI countries and 48 NPI countries. *NPI*=non-pharmaceutical interventions. Time trends of time spent in residence from February 15 to March 1^st^, 2020. Day refers to day of the year. NPI considered here is lockdown implemented within 30 days of first case detection.

Figure A2: Average daily residual of time spent in residence in NPI and non-NPI countries, February 15^th^, 2020 to March 1^st^, 2020

Note: N=132 countries; 84 non-NPI countries and 48 NPI countries. *NPI*=non-pharmaceutical interventions. Time trends of the estimated residual error terms of country fixed effect regression of time spent in residence on day from February 15^th^ to March 1^st^, 2020. Day refers to day of the year. NPI considered here is lockdown implemented within 30 days of first case detection.

Figure A3: Coefficient of interaction between NPI countries and day in regression of time spent at residence, February 15^th^, 2020 to March 1^st^, 2020

Note: N=132 countries; 84 non-NPI countries and 48 NPI countries. *NPI*=non-pharmaceutical intervention. Coefficient of NPI intervention and day interaction term with 95% confidence interval from country fixed effects regression of the time spent in residence on day identifiers, binary indicator of whether a country implemented NPI, and interaction terms of the day and NPI identifiers. Day refers to day of the year. NPI considered here is lockdown implemented within 30 days of first case detection.

Table A1: Propensity score matching results on change in mobility from the implementation of non-pharmaceutical interventions (long follow-up)

| Measure | Latest day of implementation | Category | Change in mobility (%) | Confidence Interval | R^2^ | Observations |
| --- | --- | --- | --- | --- | --- | --- |
| Lockdown | 30 | Grocery and pharmacy | -21.59** | (-30.70 - -12.47) | 0.167 | 112 |
|  |  | Parks | -13.73** | (-21.86 - -5.59) | 0.092 | 112 |
|  |  | Residential | 5.55** | (1.95 - 9.16) | 0.08 | 109 |
|  |  | Retail and recreation | -16.64** | (-24.07 - -9.20) | 0.15 | 113 |
|  |  | Transit stations | -15.08** | (-21.55 - -8.61) | 0.162 | 112 |
|  |  | Workplace | -15.47** | (-21.78 - -9.16) | 0.176 | 113 |
|  | 45 | Grocery and pharmacy | -27.61** | (-37.04 - -18.18) | 0.247 | 105 |
|  |  | Parks | -22.23** | (-30.14 - -14.32) | 0.232 | 105 |
|  |  | Residential | 8.39** | (4.68 - 12.09) | 0.168 | 102 |
|  |  | Retail and recreation | -21.45** | (-29.14 - -13.76) | 0.229 | 105 |
|  |  | Transit stations | -18.86** | (-25.58 - -12.14) | 0.231 | 105 |
|  |  | Workplace | -18.07** | (-24.62 - -11.53) | 0.225 | 105 |
| Lockdown strict | 30 | Grocery and pharmacy | -31.54** | (-41.71 - -21.36) | 0.255 | 112 |
|  |  | Parks | -18.40** | (-29.48 - -7.32) | 0.09 | 112 |
|  |  | Residential | 9.70** | (5.76 - 13.65) | 0.182 | 109 |
|  |  | Retail and recreation | -24.37** | (-32.43 - -16.30) | 0.244 | 113 |
|  |  | Transit stations | -20.76** | (-27.98 - -13.54) | 0.228 | 112 |
|  |  | Workplace | -23.71** | (-30.57 - -16.86) | 0.297 | 113 |
|  | 45 | Grocery and pharmacy | -32.70** | (-42.63 - -22.76) | 0.297 | 103 |
|  |  | Parks | -12.89** | (-21.82 - -3.96) | 0.075 | 103 |
|  |  | Residential | 9.44** | (5.61 - 13.27) | 0.193 | 102 |
|  |  | Retail and recreation | -22.67** | (-30.81 - -14.53) | 0.232 | 103 |
|  |  | Transit stations | -18.77** | (-25.95 - -11.58) | 0.21 | 103 |
|  |  | Workplace | -22.44** | (-30.18 - -14.69) | 0.246 | 103 |
| School | 30 | Grocery and pharmacy | -12.19* | (-23.31 - -1.06) | 0.049 | 94 |
|  |  | Parks | -12.39* | (-22.48 - -2.31) | 0.061 | 94 |
|  |  | Residential | 3.32 | (-1.28 - 7.91) | 0.023 | 91 |
|  |  | Retail and recreation | -6.38 | (-15.10 - 2.35) | 0.02 | 106 |
|  |  | Transit stations | -8.58* | (-16.16 - -1.01) | 0.052 | 94 |
|  |  | Workplace | -5.2 | (-13.76 - 3.37) | 0.014 | 106 |
|  | 45 | Grocery and pharmacy | -9.22 | (-20.57 - 2.13) | 0.027 | 96 |
|  |  | Parks | -7.25 | (-18.43 - 3.93) | 0.017 | 96 |
|  |  | Residential | 5.04* | (0.34 - 9.74) | 0.047 | 93 |
|  |  | Retail and recreation | -10.08* | (-19.42 - -0.73) | 0.046 | 96 |
|  |  | Transit stations | -10.72** | (-18.59 - -2.84) | 0.072 | 96 |
|  |  | Workplace | -9.20* | (-17.20 - -1.21) | 0.053 | 96 |

Note: Outcome is change in mobility from 1 day before to 6 days after intervention. Results from propensity score matching using one-to-one nearest neighbor matching and imposing common support. 95% confidence intervals in parentheses. +p<0.1, *p<0.05, **p<0.01.

Table A2: Propensity score matching results on change in mobility from the implementation of lockdown (alternate matching)

| Latest delay in implementation (in days) | Matching model | Category | Change in mobility (%) | Confidence Interval | R^2^ | Observations |
| --- | --- | --- | --- | --- | --- | --- |
| 30 | Nearest three neighbors | Grocery and pharmacy | -19.82** | (-29.12 - -10.53) | 0.14 | 112 |
|  | Kernel |  | -19.82** | (-29.12 - -10.53) | 0.14 | 112 |
|  | Nearest three neighbors | Parks | -13.96** | (-21.91 - -6.01) | 0.099 | 112 |
|  | Kernel |  | -13.96** | (-21.91 - -6.01) | 0.099 | 112 |
|  | Nearest three neighbors | Residential | 6.67** | (3.58 - 9.75) | 0.145 | 110 |
|  | Kernel |  | 6.67** | (3.58 - 9.75) | 0.145 | 110 |
|  | Nearest three neighbors | Retail and recreation | -16.25** | (-23.42 - -9.07) | 0.154 | 113 |
|  | Kernel |  | -16.25** | (-23.42 - -9.07) | 0.154 | 113 |
|  | Nearest three neighbors | Transit stations | -15.84** | (-21.55 - -10.13) | 0.215 | 112 |
|  | Kernel |  | -15.84** | (-21.55 - -10.13) | 0.215 | 112 |
|  | Nearest three neighbors | Workplace | -15.41** | (-21.62 - -9.20) | 0.179 | 113 |
|  | Kernel |  | -15.41** | (-21.62 - -9.20) | 0.179 | 113 |
| 45 | Nearest three neighbors | Grocery and pharmacy | -26.38** | (-36.16 - -16.61) | 0.218 | 105 |
|  | Kernel |  | -26.38** | (-36.16 - -16.61) | 0.218 | 105 |
|  | Nearest three neighbors | Parks | -20.06** | (-26.96 - -13.17) | 0.244 | 105 |
|  | Kernel |  | -20.06** | (-26.96 - -13.17) | 0.244 | 105 |
|  | Nearest three neighbors | Residential | 6.62** | (3.48 - 9.77) | 0.147 | 103 |
|  | Kernel |  | 6.62** | (3.48 - 9.77) | 0.147 | 103 |
|  | Nearest three neighbors | Retail and recreation | -23.21** | (-30.88 - -15.54) | 0.259 | 105 |
|  | Kernel |  | -23.21** | (-30.88 - -15.54) | 0.259 | 105 |
|  | Nearest three neighbors | Transit stations | -19.77** | (-25.87 - -13.66) | 0.286 | 105 |
|  | Kernel |  | -19.77** | (-25.87 - -13.66) | 0.286 | 105 |
|  | Nearest three neighbors | Workplace | -11.74** | (-18.07 - -5.41) | 0.116 | 105 |
|  | Kernel |  | -11.74** | (-18.07 - -5.41) | 0.116 | 105 |

Note: Outcome is change in mobility from 1 day before to 2 days after intervention. Common support imposed. 95% confidence intervals in parentheses. +p<0.1, *p<0.05, **p<0.01.

Table A3: Propensity score matching results on change in mobility from the implementation of strict lockdown (alternate matching)

| Latest delay in implementation (in days) | Matching model | Category | Change in mobility (%) | Confidence Interval | R^2^ | Observations |
| --- | --- | --- | --- | --- | --- | --- |
| 30 | Nearest three neighbors | Grocery and pharmacy | -30.98** | (-41.44 - -20.51) | 0.238 | 112 |
|  | Kernel |  | -30.98** | (-41.44 - -20.51) | 0.238 | 112 |
|  | Nearest three neighbors | Parks | -19.41** | (-28.21 - -10.61) | 0.148 | 112 |
|  | Kernel |  | -19.41** | (-28.21 - -10.61) | 0.148 | 112 |
|  | Nearest three neighbors | Residential | 9.40** | (6.08 - 12.72) | 0.227 | 109 |
|  | Kernel |  | 9.40** | (6.08 - 12.72) | 0.227 | 109 |
|  | Nearest three neighbors | Retail and recreation | -25.58** | (-33.39 - -17.76) | 0.275 | 113 |
|  | Kernel |  | -25.58** | (-33.39 - -17.76) | 0.275 | 113 |
|  | Nearest three neighbors | Transit stations | -21.70** | (-28.13 - -15.27) | 0.289 | 112 |
|  | Kernel |  | -21.70** | (-28.13 - -15.27) | 0.289 | 112 |
|  | Nearest three neighbors | Workplace | -21.69** | (-28.58 - -14.79) | 0.259 | 113 |
|  | Kernel |  | -21.69** | (-28.58 - -14.79) | 0.259 | 113 |
| 45 | Nearest three neighbors | Grocery and pharmacy | -30.65** | (-40.96 - -20.34) | 0.256 | 103 |
|  | Kernel |  | -30.65** | (-40.96 - -20.34) | 0.256 | 103 |
|  | Nearest three neighbors | Parks | -17.68** | (-25.73 - -9.62) | 0.158 | 103 |
|  | Kernel |  | -17.68** | (-25.73 - -9.62) | 0.158 | 103 |
|  | Nearest three neighbors | Residential | 10.77** | (7.19 - 14.36) | 0.262 | 102 |
|  | Kernel |  | 10.77** | (7.19 - 14.36) | 0.262 | 102 |
|  | Nearest three neighbors | Retail and recreation | -23.49** | (-31.59 - -15.38) | 0.247 | 103 |
|  | Kernel |  | -23.49** | (-31.59 - -15.38) | 0.247 | 103 |
|  | Nearest three neighbors | Transit stations | -20.88** | (-27.71 - -14.06) | 0.267 | 103 |
|  | Kernel |  | -20.88** | (-27.71 - -14.06) | 0.267 | 103 |
|  | Nearest three neighbors | Workplace | -24.67** | (-33.62 - -15.73) | 0.229 | 103 |
|  | Kernel |  | -24.67** | (-33.62 - -15.73) | 0.229 | 103 |

Note: Outcome is change in mobility from 1 day before to 2 days after intervention. Common support imposed. 95% confidence intervals in parentheses. +p<0.1, *p<0.05, **p<0.01.

Table A4: Propensity score matching results on change in mobility from the implementation of school closure (alternate matching)

| Latest delay in implementation (in days) | Matching model | Category | Change in mobility (%) | Confidence Interval | R2 | Observations |
| --- | --- | --- | --- | --- | --- | --- |
| 30 | Nearest three neighbors | Grocery and pharmacy | -7.31+ | (-15.92 - 1.30) | 0.029 | 96 |
|  | Kernel |  | -7.31+ | (-15.92 - 1.30) | 0.029 | 96 |
|  | Nearest three neighbors | Parks | -7.83* | (-15.62 - -0.03) | 0.041 | 96 |
|  | Kernel |  | -7.83* | (-15.62 - -0.03) | 0.041 | 96 |
|  | Nearest three neighbors | Residential | 3.34* | (0.18 - 6.50) | 0.046 | 94 |
|  | Kernel |  | 3.34* | (0.18 - 6.50) | 0.046 | 94 |
|  | Nearest three neighbors | Retail and recreation | -9.58** | (-16.41 - -2.76) | 0.076 | 96 |
|  | Kernel |  | -9.58** | (-16.41 - -2.76) | 0.076 | 96 |
|  | Nearest three neighbors | Transit stations | -8.57** | (-14.16 - -2.99) | 0.09 | 96 |
|  | Kernel |  | -8.57** | (-14.16 - -2.99) | 0.09 | 96 |
|  | Nearest three neighbors | Workplace | -6.94* | (-13.55 - -0.32) | 0.044 | 96 |
|  | Kernel |  | -6.94* | (-13.55 - -0.32) | 0.044 | 96 |
| 45 | Nearest three neighbors | Grocery and pharmacy | -7.03 | (-16.02 - 1.97) | 0.027 | 90 |
|  | Kernel |  | -7.03 | (-16.02 - 1.97) | 0.027 | 90 |
|  | Nearest three neighbors | Parks | -5.97 | (-14.25 - 2.31) | 0.023 | 90 |
|  | Kernel |  | -5.97 | (-14.25 - 2.31) | 0.023 | 90 |
|  | Nearest three neighbors | Residential | 2.36 | (-0.93 - 5.65) | 0.023 | 88 |
|  | Kernel |  | 2.36 | (-0.93 - 5.65) | 0.023 | 88 |
|  | Nearest three neighbors | Retail and recreation | -9.95** | (-16.94 - -2.96) | 0.083 | 90 |
|  | Kernel |  | -9.95** | (-16.94 - -2.96) | 0.083 | 90 |
|  | Nearest three neighbors | Transit stations | -7.95** | (-13.54 - -2.35) | 0.083 | 90 |
|  | Kernel |  | -7.95** | (-13.54 - -2.35) | 0.083 | 90 |
|  | Nearest three neighbors | Workplace | -5.18 | (-11.97 - 1.61) | 0.025 | 90 |
|  | Kernel |  | -5.18 | (-11.97 - 1.61) | 0.025 | 90 |

Note: Outcome is change in mobility from 1 day before to 2 days after intervention. Common support imposed. 95% confidence intervals in parentheses. +p<0.1, *p<0.05, **p<0.01.

Table A5: Balancing between matched and control groups from lockdown model

| Data | Maximum implementation delay (days) | Location | Pseudo R^2^ | P>Chi^2^ | Mean bias | Median bias |
| --- | --- | --- | --- | --- | --- | --- |
| Matched | 30 | Grocery and pharmacy | 0.11 | 0.58 | 14.99 | 12.09 |
| Unmatched |  |  | 0.28 | 0.00 | 24.80 | 23.27 |
| Matched |  | Parks | 0.11 | 0.58 | 14.99 | 12.09 |
| Unmatched |  |  | 0.28 | 0.00 | 24.80 | 23.27 |
| Matched |  | Residential | 0.43 | 0.00 | 22.74 | 21.27 |
| Unmatched |  |  | 0.28 | 0.00 | 26.45 | 25.90 |
| Matched |  | Retail and recreation | 0.11 | 0.68 | 15.01 | 13.97 |
| Unmatched |  |  | 0.28 | 0.00 | 24.60 | 23.00 |
| Matched |  | Transit stations | 0.11 | 0.58 | 14.99 | 12.09 |
| Unmatched |  |  | 0.28 | 0.00 | 24.80 | 23.27 |
| Matched |  | Workplace | 0.11 | 0.68 | 15.01 | 13.97 |
| Unmatched |  |  | 0.28 | 0.00 | 24.60 | 23.00 |
| Matched | 40 | Grocery and pharmacy | 0.10 | 0.57 | 11.33 | 9.99 |
| Unmatched |  |  | 0.27 | 0.00 | 28.03 | 28.08 |
| Matched |  | Parks | 0.10 | 0.57 | 11.33 | 9.99 |
| Unmatched |  |  | 0.27 | 0.00 | 28.03 | 28.08 |
| Matched |  | Residential | 0.13 | 0.31 | 13.83 | 13.63 |
| Unmatched |  |  | 0.27 | 0.00 | 29.40 | 32.87 |
| Matched |  | Retail and recreation | 0.10 | 0.57 | 11.33 | 9.99 |
| Unmatched |  |  | 0.27 | 0.00 | 28.03 | 28.08 |
| Matched |  | Transit stations | 0.10 | 0.57 | 11.33 | 9.99 |
| Unmatched |  |  | 0.27 | 0.00 | 28.03 | 28.08 |
| Matched |  | Workplace | 0.10 | 0.57 | 11.33 | 9.99 |
| Unmatched |  |  | 0.27 | 0.00 | 28.03 | 28.08 |

Note: Results from propensity score matching using one-to-one nearest neighbor matching and imposing common support.

Table A6: Balancing between matched and control groups from strict lockdown model

| Data | Maximum implementation delay (days) | Location | Pseudo R^2^ | P>Chi^2^ | Mean bias | Median bias |
| --- | --- | --- | --- | --- | --- | --- |
| Matched | 30 | Grocery and pharmacy | 0.13 | 0.83 | 15.43 | 14.33 |
| Unmatched |  |  | 0.21 | 0.03 | 22.01 | 20.10 |
| Matched |  | Parks | 0.13 | 0.83 | 15.43 | 14.33 |
| Unmatched |  |  | 0.21 | 0.03 | 22.01 | 20.10 |
| Matched |  | Residential | 0.21 | 0.42 | 13.63 | 10.04 |
| Unmatched |  |  | 0.21 | 0.04 | 22.18 | 19.18 |
| Matched |  | Retail and recreation | 0.05 | 0.99 | 14.54 | 15.24 |
| Unmatched |  |  | 0.21 | 0.03 | 21.15 | 18.36 |
| Matched |  | Transit stations | 0.13 | 0.83 | 15.43 | 14.33 |
| Unmatched |  |  | 0.21 | 0.03 | 22.01 | 20.10 |
| Matched |  | Workplace | 0.05 | 0.99 | 14.54 | 15.24 |
| Unmatched |  |  | 0.21 | 0.03 | 21.15 | 18.36 |
| Matched | 45 | Grocery and pharmacy | 0.20 | 0.24 | 16.66 | 14.96 |
| Unmatched |  |  | 0.17 | 0.10 | 22.73 | 20.18 |
| Matched |  | Parks | 0.20 | 0.24 | 16.66 | 14.96 |
| Unmatched |  |  | 0.17 | 0.10 | 22.73 | 20.18 |
| Matched |  | Residential | 0.11 | 0.75 | 15.09 | 13.91 |
| Unmatched |  |  | 0.17 | 0.11 | 22.56 | 20.59 |
| Matched |  | Retail and recreation | 0.20 | 0.24 | 16.66 | 14.96 |
| Unmatched |  |  | 0.17 | 0.10 | 22.73 | 20.18 |
| Matched |  | Transit stations | 0.20 | 0.24 | 16.66 | 14.96 |
| Unmatched |  |  | 0.17 | 0.10 | 22.73 | 20.18 |
| Matched |  | Workplace | 0.20 | 0.24 | 16.66 | 14.96 |
| Unmatched |  |  | 0.17 | 0.10 | 22.73 | 20.18 |

Note: Results from propensity score matching using one-to-one nearest neighbor matching and imposing common support.

Table A7: Balancing between matched and control groups from school closure model

| Data | Maximum implementation delay (days) | Location | Pseudo R^2^ | P>Chi^2^ | Mean bias | Median bias |
| --- | --- | --- | --- | --- | --- | --- |
| Matched | 30 | Grocery and pharmacy | 0.14 | 0.23 | 14.91 | 13.65 |
| Unmatched |  |  | 0.57 | 0.00 | 44.74 | 37.78 |
| Matched |  | Parks | 0.14 | 0.23 | 14.91 | 13.65 |
| Unmatched |  |  | 0.57 | 0.00 | 44.74 | 37.78 |
| Matched |  | Residential | 0.16 | 0.18 | 14.40 | 13.42 |
| Unmatched |  |  | 0.57 | 0.00 | 44.99 | 37.15 |
| Matched |  | Retail and recreation | 0.10 | 0.34 | 15.01 | 13.48 |
| Unmatched |  |  | 0.48 | 0.00 | 43.66 | 41.72 |
| Matched |  | Transit stations | 0.14 | 0.23 | 14.91 | 13.65 |
| Unmatched |  |  | 0.57 | 0.00 | 44.74 | 37.78 |
| Matched |  | Workplace | 0.10 | 0.34 | 15.01 | 13.48 |
| Unmatched |  |  | 0.48 | 0.00 | 43.66 | 41.72 |
| Matched | 40 | Grocery and pharmacy | 0.33 | 0.00 | 26.36 | 25.57 |
| Unmatched |  |  | 0.59 | 0.00 | 56.25 | 55.75 |
| Matched |  | Parks | 0.33 | 0.00 | 26.36 | 25.57 |
| Unmatched |  |  | 0.59 | 0.00 | 56.25 | 55.75 |
| Matched |  | Residential | 0.20 | 0.01 | 22.30 | 15.80 |
| Unmatched |  |  | 0.58 | 0.00 | 56.50 | 55.11 |
| Matched |  | Retail and recreation | 0.33 | 0.00 | 26.36 | 25.57 |
| Unmatched |  |  | 0.59 | 0.00 | 56.25 | 55.75 |
| Matched |  | Transit stations | 0.33 | 0.00 | 26.36 | 25.57 |
| Unmatched |  |  | 0.59 | 0.00 | 56.25 | 55.75 |
| Matched |  | Workplace | 0.33 | 0.00 | 26.36 | 25.57 |
| Unmatched |  |  | 0.59 | 0.00 | 56.25 | 55.75 |

Note: Results from propensity score matching using one-to-one nearest neighbor matching and imposing common support.
